# Molecular chaperones and protein folding as therapeutic targets in Parkinson’s disease and other synucleinopathies

**DOI:** 10.1186/2051-5960-1-79

**Published:** 2013-12-05

**Authors:** Darius Ebrahimi-Fakhari, Laiq-Jan Saidi, Lara Wahlster

**Affiliations:** Division of Neurology & Division of Inherited Metabolic Diseases, Department of Pediatrics I, Children’s Hospital, Heidelberg University Hospital, Ruprecht-Karls-University Heidelberg, INF 430, 69120 Heidelberg, Germany; Neuroscience Program, Faculty of Medicine and Faculty of Mathematics & Natural Sciences, University of Cologne, Cologne, Germany

**Keywords:** Neurodegeneration, Parkinson’s disease, Alpha-synuclein, Molecular chaperone, Heat shock protein, Hsp70, Hsp90, Proteasome, Autophagy, Apoptosis

## Abstract

Changes in protein metabolism are key to disease onset and progression in many neurodegenerative diseases. As a prime example, in Parkinson’s disease, folding, post-translational modification and recycling of the synaptic protein α-synuclein are clearly altered, leading to a progressive accumulation of pathogenic protein species and the formation of intracellular inclusion bodies. Altered protein folding is one of the first steps of an increasingly understood cascade in which α-synuclein forms complex oligomers and finally distinct protein aggregates, termed Lewy bodies and Lewy neurites. In neurons, an elaborated network of chaperone and co-chaperone proteins is instrumental in mediating protein folding and re-folding. In addition to their direct influence on client proteins, chaperones interact with protein degradation pathways such as the ubiquitin-proteasome-system or autophagy in order to ensure the effective removal of irreversibly misfolded and potentially pathogenic proteins. Because of the vital role of proper protein folding for protein homeostasis, a growing number of studies have evaluated the contribution of chaperone proteins to neurodegeneration. We herein review our current understanding of the involvement of chaperones, co-chaperones and chaperone-mediated autophagy in synucleinopathies with a focus on the Hsp90 and Hsp70 chaperone system. We discuss genetic and pathological studies in Parkinson’s disease as well as experimental studies in models of synucleinopathies that explore molecular chaperones and protein degradation pathways as a novel therapeutic target. To this end, we examine the capacity of chaperones to prevent or modulate neurodegeneration and summarize the current progress in models of Parkinson’s disease and related neurodegenerative disorders.

## Introduction

Parkinson’s disease (PD) is a common incurable neurodegenerative disease that affects around 1% of the worldwide population at age 60 years [1]. It is progressive in nature and causes a movement disorder characterized by bradykinesia, resting tremor, rigidity and postural instability along with non-motor symptoms that mainly include autonomic dysfunction and cognitive impairment [2]. No treatment with established efficacy in preventing or slowing the progression of neurodegeneration in PD is currently available and development of such treatment is of utmost importance. Progressive degeneration of neurons in defined regions of the brain and the presence of proteinaceous intracellular inclusion bodies characterize PD pathology [3]. These inclusion bodies are termed Lewy bodies and Lewy neurites and contain large amounts of ubiquitinated and phosphorylated proteins, most importantly the presynaptic protein α-synuclein [3–5]. Increased levels of α-synuclein or α-synuclein containing protein aggregates are not only a hallmark of PD but are characteristic for a whole group of neurodegenerative diseases including dementia with Lewy bodies (DLB), multiple system atrophy (MSA), Alzheimer’s disease, different forms of neurodegeneration with brain iron accumulation and others [3, 6–8]. This group of diseases can therefore be referred to as “synucleinopathies”, although overlapping pathologies (such as tau-containing neurofibrillary tangles or amyloid-β plaques) exist in many cases and may act synergistically. Strong evidence for an involvement of α-synuclein in PD is also provided by genetic studies in familial and sporadic forms of the disease. Missense mutations in the α-synuclein gene (*SCNA*) (A53T, A30P and E46K) [9–11] as well as gene multiplications [12–14] cause familial forms of PD, while recent genome wide association studies have revealed polymorphisms in the α-synuclein gene as risk factors for developing sporadic PD [15].

An emerging theme in many neurodegenerative diseases, including the synucleinopathies, are deficits in protein metabolism, most importantly protein folding and degradation [16–23]. Alpha-synuclein is a neuronal protein that is enriched at presynaptic terminals, where it is thought to be involved in the assembly of the SNARE (soluble NSF attachment protein receptor) machinery and vesicle release [24, 25]. Alpha-synuclein pathology in PD is believed to follow a multi-step process that starts with the misfolding of α-synuclein and progresses to the formation of increasingly complex oligomers, soluble intermediates and finally insoluble fibrils and mature aggregates [17–19]. Although α-synuclein has been classically described to have an unfolded tertiary structure and to be present as monomers that acquire an α-helical secondary structure upon binding to lipid membranes [26, 27], recent reports suggest that α-synuclein natively forms α-helically folded tetramers when isolated under non-denaturing conditions [28–30]. These results have a significant impact on future research because they add a new step to the sequence of pathological events in synucleinopathies: Events that destabilize the native α-helical tetramer conformation might precede α-synuclein misfolding and aggregation and thus compounds that preserve the native tetramers may have great therapeutic potential. It should be cautioned however that experiments from two independent laboratories have failed to confirm the presence of natively unfolded α-synuclein tetramers in PD [31, 32]. Future studies will have to decipher the exact mechanisms behind these findings and will have to explain conflicting results.

Moving downstream of simple α-synuclein misfolding, emerging evidence implicates soluble oligomeric forms of α-synuclein as the main culprit in the pathogenesis of neurodegenerative diseases associated with α-synuclein accumulation [19]. Disease causing missense mutations and multiplications of the α-synuclein gene [33] as well as oxidative stress [34], post-translational modifications such as phosphorylation [35, 36] or truncation [37, 38] and the presence of fatty acids [39–41] are known to modulate α-synuclein’s propensity to aggregate. Furthermore, levels of α-synuclein oligomers are increased in cortical tissue of patients with idiopathic PD [40] and DLB [42] compared to age-matched controls. The mechanism by which smaller soluble aggregates induce neuronal dysfunction and neurodegeneration is increasingly, albeit still incompletely, understood [19]. Using a protein-fragment complementation assay in transfected cells and viral-vector mediated rodent models of α-synuclein aggregation, oligomer formation was shown to contribute to α-synuclein’s toxic effect on neurons [43–48]. Importantly, α-synuclein oligomers are involved in key steps of the potentially prion-like propagation of neurodegeneration in PD such as exocytosis, endocytosis and seeding [19, 49–51]. Given the implications of α-synuclein oligomerization in the early stages of neurodegeneration, preventing this step is a promising approach to treat or even prevent the degenerative process associated with α-synuclein misfolding and accumulation.

## Review

### Molecular chaperones, co-chaperones and chaperone-mediated autophagy

A network of highly conserved molecules, termed chaperones and co-chaperones, mediates the folding and re-folding of proteins and thus is critical for preserving the functional state and structure of client proteins [52–55]. Molecular chaperones are defined as a class of proteins that interact with, stabilize and help proteins to acquire their native conformation [52]. They are highly ubiquitous and assist the folding of newly synthesized proteins as well as the refolding of partially folded proteins into their three-dimensional structures [52, 53, 56]. In order to preserve intracellular protein homeostasis, chaperones interact with pathways of protein degradation that regulate constitutive protein turnover and the removal of misfolded proteins. Major protein degradation pathways for α-synuclein are the ubiquitin-proteasome system and the autophagy-lysosomal pathway [18, 57]. According to their molecular weight, chaperones can be classified into different groups such as Hsp60, Hsp70, Hsp90, Hsp100 and the small Hsps. Important co-chaperones, which interact with and assist chaperones in the folding of their client proteins, include for example the BAG-domain containing family (Bag1-6), the TPR-domain containing family (CHIP, Hip, Hop) and the DnaJ-domain containing co-chaperone Hsp40 [17, 22]. Cells constitutively express many chaperones (then referred to as heat shock cognates (*Hsc*)) and co-chaperones. However, their expression is markedly increased under environmental stress conditions, for example following hyperthermia, hypoxia, oxidative stress or exposure to toxins [52–54, 56, 58]. This stress response is triggered by the accumulation of unfolded proteins and effectively elicits chaperone expression by a signaling pathway that engages the transcription factor heat shock factor 1 (HSF-1) [54, 59, 60]. This regulatory element is part of a molecular switch that adjusts levels of chaperones to the cell’s condition. Hsp90 associates with HSF-1 in the cytosol and thus preserves its inactive monomeric state [61]. Cell stress and protein misfolding promote the dissociation of HSF-1 from Hsp90 and hence its translocation to the nucleus. At the nucleus, HSF-1 initiates the coordinated expression of Hsp70 and other heat shock proteins via heat shock response elements in the promoter regions of the respective genes [62]. Once adequate levels of chaperones have reached the cytosol, Hsp90 again associates with and inactivates HSF-1 therefore creating a dynamic Hsp90-dependent feedback loop that allows the cell to adjust to endogenous or exogenous stress [63, 64]. This feedback loop also opens opportunities to pharmacologically modulate chaperone levels, or levels of Hsp70 in particular, by applying inhibitors of Hsp90, a concept that is being increasingly investigated [17, 22].

In addition to directly folding or re-folding substrate proteins, chaperones assist many other cellular pathways for example by selecting and targeting irreversibly damaged or altered proteins for degradation. Chaperone-mediated autophagy refers to a highly-selective subtype of autophagy that utilizes chaperone proteins and lysosomal receptors to directly translocate target proteins into the lysosomal lumen, where rapid degradation takes place [65]. Target proteins carry a pentapeptide motif (KFERQ) and are thus selectively identified by the cytosolic chaperone Hsc70, a constitutively expressed member of the Hsp70 family, that facilitates delivery to the lysosomal surface [66–68]. The action of Hsc70 and its co-chaperones is crucial as the interaction with the KFERQ targeting motif confers selectivity. At the lysosomal membrane, binding of the substrate-chaperone complex to the lysosomal receptor protein LAMP-2A is followed by unfolding, multimerization of LAMP-2A, and finally translocation of the target protein [68, 69]. Lysosome-associated Hsc70, that resides within the lysosomal lumen, assists the disassembly of the LAMP-2A multimer complex after translocation and thus regenerates monomeric forms of LAMP-2A, that are again capable of substrate binding [70, 71]. The presence of lysosomal Hsc70 is a critical rate-limiting step, as, although all types of lysosomes carry the LAMP-2A receptor, only lysosomes that contain lysosomal Hsc70 show effective substrate uptake [72]. Interestingly, another chaperone, Hsp90 localizes to both the cytosolic and luminal side of the lysosomal membrane and is thought to stabilize LAMP-2A as it transitions from its monomeric form capable of substrate binding to the multimeric form that allows substrate translocation across the membrane [71]. The wide spectrum of cellular functions in which CMA is critically involved, ranging from selective protein quality control to cell-type specific functions depending on the substrate protein, emphasizes the importance of this pathway for maintaining protein homeostasis and cellular integrity, particularly in response to stress. CMA activity declines with age in many tissues [73, 74] and failure of CMA has been linked to the pathogenesis of several major neurodegenerative diseases, including the synucleinopathies (as discussed below).

### Chaperones protect neurons against α-synuclein-induced toxicity

**Figure 1:**
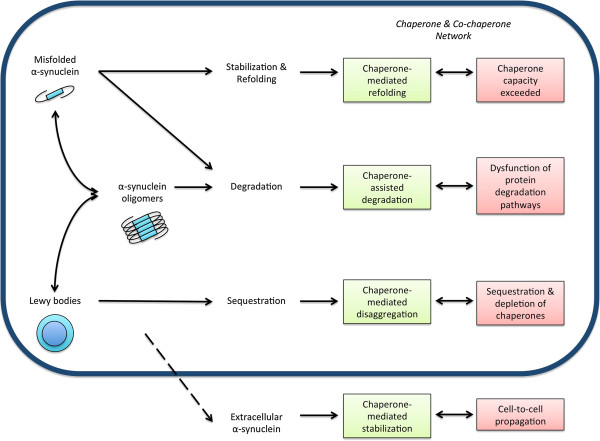
**The role of chaperones and co-chaperones in α-synuclein metabolism and pathology.** As a general concept, chaperones mediate several cellular strategies that maintain protein homeostasis. In synucleinopathies, misfolded α-synuclein can be refolded, degraded, secreted or sequestered into mature aggregates such as Lewy bodies. Direct stabilization and refolding, degradation via different protein degradation pathways and sequestration into aggregates are mechanisms that are assisted or modulated by chaperones and co-chaperones. Failure of these mechanisms abolishes protein homeostasis and thus promotes α-synuclein accumulation, oligomer formation, toxicity and potentially cell-to-cell propagation of α-synuclein pathology.

Research investigating the role of molecular chaperones in synucleinopathies followed groundbreaking work in other neurodegenerative diseases, most importantly the trinucleotide repeat expansions disorders [75–78]. First evidence for an involvement of chaperones in PD was provided by studies that identified Hsp90, Hsp70, Hsp60, Hsp40 and Hsp27 as part of Lewy bodies [79–82]. In a seminal study, *Auluck et al.* were able to demonstrate that Hsp70 co-expression could prevent dopaminergic cell death in a *Drosophila melanogaster* model of α-synuclein toxicity [81]. Furthermore interference with the endogenous chaperone system by introducing a mutation to Hsp70 could exacerbate the pathological phenotype, confirming the notion that Hsp70 is critical for maintaining α-synuclein’s folding state [81]. Based on these initial findings two pivotal hypotheses have been formulated and investigated in subsequent studies (reviewed in [17]). Firstly, Hsp70 is a critical part of the cellular mechanism that mitigates α-synuclein toxicity and secondly the sequestration of chaperones into protein aggregates results in their cellular depletion and thus subsequent loss of chaperone function may promote neurodegeneration (Figure 1).

Consistent with the idea that chaperones are a critical part of the response to environmental stress and protein overload, cells [83] and mice [84] treated with the mitochondrial toxins rotenone or MPTP (1-methyl-4-phenyl-1,2,3,6-tetrahydropyridine) or the proteasome inhibitor lactacystin, which are often used to model dopaminergic cell degeneration, show a marked increase in chaperone levels, most importantly Hsp70. Likewise viral-vector mediated targeted overexpression of α-synuclein in the substantia nigra of mice resulted in increased mRNA levels of Hsp70, Hsp40 and Hsp27 [85]. An interesting recent study by *Donmez et al.* reported that SIRT1, a member of the sirtuin protein deacetylase family, deacetylates HSF-1 in the brain of A53T mutant α-synuclein mice, thus promoting the expression of Hsp70 [86]. This suggests that SIRT1 deacetylates HSF-1 and activates chaperones under stress conditions induced by the presence of mutant α-synuclein. Subsequently this mechanism leads to a suppression of α-synuclein aggregation, reduced α-synuclein-induced toxicity and extended survival in the mouse model examined [86].

Critical to novel therapeutic strategies, exogenous overexpression of Hsp70 and other chaperones has proven neuroprotective in different PD models. In cell culture models of α-synuclein aggregation and toxicity, co-expression of TorsinA (a protein with homology to Hsp104) [79], Hsp40 [79, 87], Hsp27 [88, 89], or Hsp70 [90, 91] led to reduced aggregate formation, decreased α-synuclein levels and reduced toxicity (Figure 1).

Despite these promising findings, studies evaluating different chaperones as a target of therapy in mouse models of PD provided differing results. While *Klucken et al.* showed that crossing of Hsp70 transgenic mice with α-synuclein transgenic mice reduced α-synuclein aggregation *in vivo*[91], *Shimshek et al.* could not confirm this finding after crossing human A53T mutant α-synuclein transgenic mice with mice overexpressing Hsp70 [92]. This argues that frank overexpression of Hsp70 alone might not have a significant impact on α-synuclein-induced toxicity *in vivo*. Similarly, while TorsinA was found to be a potent suppressor of α-synuclein aggregation and toxicity in cellular models [79] and in a *Caenorhabditis elegans* model [93], an elegant recent study using both an MPTP-induced mouse model of PD and α-synuclein transgenic mice could not detect a neuroprotective effect for overexpression of TorsinA [94].

Deciphering the molecular interaction between Hsp70 and α-synuclein, Hsp70 was found to bind α-synuclein fibrils with great affinity, through a transient and reversible interaction of Hsp70’s substrate-binding domain and the core hydrophobic region of soluble α-synuclein intermediates [95, 96]. A recent study was further able to map the exact Hsc70-α-synuclein interface, which might allow the development of an Hsc70-derived polypeptide that mimics the effects of this chaperone on α-synuclein assembly and toxicity [97]. Hsp70 was shown to promote an open conformational state that discourages interaction with other α-synuclein molecules and thus the formation of oligomers [43, 98]. Furthermore, oligomer formation of secreted extracellular α-synuclein was significantly reduced when Hsp70 was co-expressed and potentially simultaneously secreted [46], a finding that might have great implications for the propagation of α-synuclein pathology and neurodegeneration (Figure 1). A systematic investigation of the interaction of various small Hsps (αB-crystallin, Hsp27, Hsp20, HspB8, and HspB2B3) with both wild-type and mutant α-synuclein showed that all small Hsps transiently bind to the various forms of α-synuclein and inhibit mature α-synuclein fibril formation [99]. Further *in vitro* characterization showed that the small Hsp HspB5 can potentiate α-synuclein fiber depolymerization by several chaperones including Hsp70 and its co-chaperones [100]. Interestingly, Hsp90 has been shown to be a critical modulator of α-synuclein aggregation [101] and can bind A53T mutant α-synuclein oligomers in an ATP-independent manner to form a stable complex, thus rendering them non-toxic to cells [102].

### Sequestration and depletion of chaperones into intracellular protein aggregates exacerbates neurodegeneration

Central to the idea that sequestration of chaperones into protein aggregates could result in a significant depletion is the finding that chaperone activity as well as the cell’s resistance to proteotoxic insults declines with age [18, 20] (Figure 1). This goes hand in hand with an increase in proteotoxic stress load over the lifetime of a cell, which is particularly important for post-mitotic cells like neurons [103]. As for chaperone sequestration in the PD brain, post-mortem pathological studies demonstrate, for example, the presence of αB-crystallin and Hsp27 positive neurons in PD patients but not in matched controls [104, 105]. The distribution of αB-crystallin positive neurons followed a distinct pattern and greatly overlapped with Lewy body pathology, although αB-crystallin accumulation was not exclusive to Lewy body bearing neurons [105]. Interestingly, by using a series of *in vitro* techniques, *Waudby et al.* were able to show that αB-crystallin binds along the length of α-synuclein fibrils thereby inhibiting further growth and shifting the monomer-fibril equilibrium in favor of dissociation [106]. This might explain the presence of chaperones in α-synuclein containing protein inclusions and could represent a way by which this and other chaperones limit the onset and progression of protein misfolding diseases [106]. As discussed above, a number of studies have revealed an association of several chaperones with α-synuclein pathology, thus promoting the idea that chaperones are key players in PD [79–82]. Following these reports, a number of studies have measured levels of chaperones in different brain regions in synucleinopathies. Overall, these studies revealed a correlation between levels of chaperones and detergent-soluble α-synuclein [80, 88, 107–110], consistent with data that show that chaperones mainly interact with this fraction of α-synuclein. Recent findings also suggest that Hsc70 and other proteins involved in CMA or lysosomal targeting and degradation, are significantly altered in different brain regions in PD and DLB [109, 111–115] (reviewed in [18]), supporting the concept of chaperone dysfunction in synucleinopathies (Figure 1). On a molecular level, α-synuclein oligomers were found to be capable of inhibiting the Hsp70/Hsp40 system by interacting with J-domain co-chaperones [116].

### Chaperone-mediated autophagy – a link between protein-folding and degradation with implications for synucleinopathies

As discussed above, CMA is a subtype of autophagy and as such participates in the selective turnover of target proteins that contain KFERQ or KFERQ-like motifs including α-synuclein [18, 65]. Although soluble wild-type α-synuclein is a substrate of CMA [117, 118], pathogenic species of α-synuclein, such as A53T and A30P mutant α-synuclein, were found to fail translocation through the lysosomal membrane and furthermore impair degradation of other CMA substrates by binding LAMP-2A [117, 119]. Important to sporadic PD, dopamine modified wild-type α-synuclein inhibited CMA in a similar way [120]. Intriguingly, the turnover of the neuronal transcription factor MEF2D was found to depend on CMA, which was significantly disrupted by the presence of wild-type and mutant α-synuclein, leading to impaired MEF2D signaling and neurodegeneration [121]. Rat and mouse α-synuclein, containing the A53T substitution seen in familial forms of PD [122], are degraded by CMA [117, 118, 123], although this seems incongruent with findings for human A53T mutant α-synuclein [117]. Serine129 phosphorylated α-synuclein and α-synuclein oligomers are not degraded by CMA [120]. *In vivo*, α-synuclein transgenic mice were found to upregulate LAMP-2A, providing evidence that CMA is part of the stress response in synucleinopathies [123]. In post-mortem pathological studies, levels of CMA adapter proteins were found to be altered in both PD [109] and DLB [114, 115]. In addition, decreased levels of CMA proteins LAMP-2A and Hsc70 in PD brain samples were found to be secondary to deregulation of several microRNAs that regulate LAMP-2A and Hsc70 expression [124]. Providing further insights into the role of CMA in synucleinopathies, *Malkus and Ischiropoulos* recently showed that regional deficits in CMA might underlie α-synuclein aggregation and neurodegeneration in the human A53T α-synuclein transgenic mouse model [125]. CMA activity was significantly decreased in aggregation-prone regions compared to other brain regions less affected by α-synuclein pathology. Upregulation of LAMP-2A occurred in regions with developing α-synuclein inclusion bodies although this dynamic transient response was not proportional to substrate uptake or degradation [125]. Exploring the therapeutic potential of CMA in synucleinopathies, *Xilouri et al.* recently showed that overexpression of LAMP-2A in cell models leads to increased CMA and protection from α-synuclein-induced degeneration [126]. Interestingly, this protective effect was present even when steady-state levels of α-synuclein were unchanged, suggesting that mitigating α-synuclein induced CMA dysfunction mainly accounts for the protective properties [126]. *In vivo*, viral vector-mediated co-overexpression of LAMP-2A in the substantia nigra of the AAV-mediated α-synuclein overexpression mouse model of PD completely preserved nigral tyrosine hydroxylase positive neurons and restored striatal levels of dopamine [126]. Collectively, these findings highlight the important role of CMA in synucleinopathies and the potential of modulating CMA as a novel therapeutic approach.

### Chaperones, endoplasmic reticulum stress and apoptosis – implications for neuroprotection in synucleinopathies

Chaperones might protect neurons by mechanisms unrelated to their chaperone function, for example by regulating key steps in programmed cell death pathways. Programmed cell death is an umbrella term that includes apoptosis (or type I cell death) and autophagic cell death (or type II cell death), both of which are implicated in progressive neurodegenerative diseases such as PD [127]. The intrinsic or mitochondrial pathway of apoptosis is of particular importance to neurodegeneration. In this pathway three distinct phases can be delineated [128]. In the pre-mitochondrial initiation phase, cells recognize danger signals and respond by activating death-inducing pathways but also pro-survival signals in an attempt to fight cellular stressors. This is followed by the integration or mitochondrial phase, in which pro- and anti-apoptotic cascades converge on mitochondria. When pro-apoptotic signals dominate, mitochondrial membrane permeabilization follows, leading to cell death if a critical number of mitochondria are affected. In the execution or post-mitochondrial phase, mitochondrial membrane permeabilization results in the breakdown of the mitochondrial transmembrane potential, respiratory chain uncoupling, ATP depletion, generation of reactive oxygen species, the release of pro-apoptotic proteins into the cytosol and finally cell death.

Along with the mitochondrial pathway of apoptosis, chaperones, such as Hsp27, Hsp70 and Hsp90, are induced in response to various cellular stressors for example DNA damage, growth factor withdrawal, hypoxia or cytotoxic drugs [128, 129]. Several chaperones have been shown to prevent apoptosis by interfering with key regulatory proteins at different stages of the mitochondrial pathway of apoptosis (see [129–131] for a detailed review). This occurs for example by inhibiting the translocation of the pro-apoptotic protein Bax to the mitochondrial membrane and subsequent prevention of membrane permeabilization and cytochrome c release, the central phenomenon in the mitochondrial apoptosis pathway [132, 133]. Other mechanisms include direct association with Apaf-1 (apoptotic peptidase activating factor 1) by Hsp70 [134–137], blockage of AIF (apoptosis inducing factor) mitochondrial release and nuclear import [136, 138–140], interaction with cytochrome c [141] or inhibition of cathepsin release from lysosomes [142].

With regard to neurotoxin-induced models of neurodegeneration, toxic effects of rotenone and MPTP were significantly ameliorated following a transient heat-shock induced overexpression of chaperones [143–145], overexpression of Hsp70 [146] or cell-penetrating peptide (TAT-Hsp70) mediated delivery of Hsp70 in cells and mice [147]. Similarly, overexpression of Hsp27 reduced 6-hydroxydopamine induced cytochrome c release and apoptosis in dopaminergic cells [148].

In addition to their influence on mitochondrial apoptosis signaling, chaperones play a pivotal role in the endoplasmic reticulum (ER)-associated stress response. Disturbance of ER function caused by dysfunction of the ubiquitin-proteasome system and/or the accumulation of misfolded proteins leads to an evolutionary conserved stress response, termed unfolded protein response (UPR) (see [149–151] for a review). This involves a global suppression of protein synthesis and the expression of specific proteins, including ER associated chaperones such as the glucose-regulated protein 78 (Grp78/Bip), in an attempt to promote cell survival. However, if protein accumulation and ER dysfunction are severe, apoptosis will be eventually triggered [152]. Important to synucleinopathies, activation of the UPR seems to be an early event in the pathogenesis of PD [153, 154] and MSA [155], a finding that can be recapitulated in diseases models *in vitro* and *in vivo*[156–161]. *Hoozemans et al.* found increased immunoreactivity for UPR markers, phosphorylated pancreatic-like ER kinase (PERK) and eukaryotic translation initiation factor 2α (eIF2α), in neuromelanin containing dopaminergic neurons in the substantia nigra pars compacta of post-mortem PD brain samples [153]. In addition, phosphorylated PERK co-localized with increased α-synuclein immunoreactivity in dopaminergic neurons [153]. This is in agreement with increased UPR activation in models of increased A53T mutant [156, 160] or wild-type [157, 159] and phosphorylated α-synuclein [158] expression. The ER-associated chaperone and member of the heat shock protein 70 family, Grp78/BIP is at the forefront of regulating the UPR pathways. When misfolded proteins accumulate within the ER, Grp78/Bip dissociates from the three major ER stress receptors (PERK, activating transcription factor 6 (ATF6) and inositol-requiring enzyme 1 (IRE1)) capable of initiating the UPR. In agreement with the finding that Grp78/Bip binds accumulating misfolded proteins in the ER, several studies found that Grp78/Bip forms a complex with α-synuclein in cell and animal models showing α-synuclein accumulation [159–161]. This underscores the important role of this ER chaperone in the response to increased α-synuclein misfolding and aggregation. Using A53T α-synuclein transgenic mice, *Colla et al.* were further able to show that α-synuclein accumulates in the ER, induces ER chaperones and sensitizes neuronal cell to ER stress induced cell death [160]. In a second elegant study, *Colla et al.* found that toxic α-synuclein oligomers form within the ER lumen and thus might compromise the integrity of ER membranes, hence leading to chronic ER stress [162]. Exploring the therapeutic implications of attenuating ER stress, treatment of A53T α-synuclein mice and a viral-vector mediated rat of α-synucleinopathy with Salubrinal, a pharmacological inhibitor of ER stress induced toxicity, dramatically delayed the onset of motoric symptoms and decreased accumulation of α-synuclein oligomers *in vivo*. Further exploring the ER-associated chaperone Grp78/Bip as a therapeutic target, *Gorbatyuk et al.* recently showed that overexpression of this chaperone in the substantia nigra of a viral-vector mediated rat model of synucleinopathy attenuated α-synuclein-induced neurotoxicity by reducing ER stress mediators [161].

### Modulation of molecular chaperones as a novel therapeutic target in synucleinopathies

Development of neuroprotective therapies for PD and other synucleinopathies is challenging because of the slow progressive nature of these diseases, the lack of reliable biomarkers for early disease detection or disease progression and limitations of available animal models. While the available symptomatic treatment for PD patients can substantially improve motor symptoms and quality of life, there is currently no therapeutic approach that can halt or reverse neuronal degeneration in PD and other synucleinopathies. Promising novel treatment strategies that were successfully identified and evaluated in pre-clinical models include cell-based therapies (reviewed in [163]) and compounds that target different cellular pathways including mitochondrial dysfunction (reviewed in [164]), mechanisms of oxidative stress, glutamate excitotoxicity and trophic factors (reviewed in [165]) as well as altered protein metabolism (reviewed in [18]). These targets are important to many neurodegenerative diseases and research efforts will therefore not only serve patients with PD but also patients who suffer from other major diseases such as DLB, Alzheimer’s disease or Huntington’s disease. Targets in protein metabolism include misfolding and aggregation, post-translational modification and protein degradation pathways such as the ubiquitin-proteasome system and autophagy [16–18, 21, 22]. Molecular chaperones are crucially involved in protein folding and refolding and thus are promising targets that have the potential to alter early pathological changes in synucleinopathies, potentially even before significant neurodegeneration has occurred. The Hsp70 system, in particular, has emerged as a promising new target to prevent or even reverse protein misfolding and associated toxicity.

A growing number of preclinical studies have employed pharmacological compounds to upregulate chaperone expression and/or function [see [17, 22] for a detailed review]. Testing of chaperone-based therapies is not limited to PD but has been greatly influenced by research in related diseases, most importantly the trinucleotide-repeat expansion diseases [166]. Based on similarities between disease models and mechanisms, many of the compounds tested in other diseases might be promising candidates for synucleinopathies [17]. Pharmacological agents targeting molecular chaperones have mainly focused on the Hsp70 system and are categorized into three groups according to their mechanism of action: A) Hsp90 inhibitors, B) modulators of HSF-1 and C) chemical chaperones (Table 1).

**Table 1:** **Pharmacological targeting of molecular chaperones in models of synucleinopathies**

A) HSP90 inhibitors			
Compound	Disease model	Readout	Reference
Geldanamycin	Drosophila melanogaster	• Hsp70 levels	*Auluck et al. 2002*[167]
		• Toxicity	
	Cell model	• α-synuclein aggregation	*McLean et al. 2004*[168]
		• α-synuclein and chaperone levels	
		• Toxicity	
	Drosophila melanogaster	• α-synuclein aggregation	*Auluck 2005 et al.*[169]
		• Hsp70 levels	
		• Toxicity	
	Saccharomyces cerevisiae	• Oxidative stress	*Flower et al. 2005*[170]
		• Cytochrome c release	
	Cell model	• Intracellular and extracellular α-synuclein levels	*Liu et al. 2009*[171]
		• Neurite length	
		• Toxicity	
	Cell model	• α-synuclein aggregation	*Emmanouilidou et al. 2010*[172]
		• Proteasome activity	
		• Levels of poly-ubiquitinated proteins	
17-AAG	Cell model	• Extracellular α-synuclein oligomers	*Danzer et al. 2011*[46]
		• Extracellular α-synuclein and Hsp70 levels	
	Cell model	• α-synuclein oligomers	*Putcha et al. 2010*[45]
		• α-synuclein and Hsp70 levels	
		• Toxicity	
	Cell model	• α-synuclein aggregation	*Riedel et al. 2010*[173]
		• Chaperone levels	
		• Macroautophagy markers	
		• Toxicity	
SNX compounds	Cell model	• α-synuclein oligomers	*Putcha et al. 2010*[45]
		• α-synuclein and Hsp70 levels	
		• Toxicity	
**B) Enhancers of HSF-1**			
**Compound**	**Disease model**	**Readout**	**Reference**
Carbenoxolone	Cell model	• α-synuclein aggregation	*Kilpatrick et al. 2013*[174]
		• α-synuclein and chaperone levels	
		• HSF-1 localization	
**C) Chemical chaperones**			
**Compound**	**Disease model**	**Readout**	**Reference**
Trehalose	Cell model	• α-synuclein levels	*Sarkar et al. 2007*[175]
		• Macroautophagy markers	
	In vitro assays	• α-synuclein aggregation	*Yu et al. 2012*[176]
Mannitol	In vitro assays, Drosophila melanogaster, α-synuclein transgenic mice	• α-synuclein aggregation	*Shaltiel-Karyo et al. 2013*[177]
		• α-synuclein and Hsp70 levels	
		• Behavioral deficits	
		• Toxicity	
Mannosylglycerate	Saccharomyces cerevisiae	• α-synuclein aggregation	*Faria et al. 2013*[178]
		• α-synuclein and chaperone levels	
		• Toxicity	
4-phenylbutyrate	α-synuclein transgenic mice	• Phosphorylated α-synuclein	*Ono et al. 2009*[179]
		• Dopamine levels	
		• Behavioral deficits	
		• Toxicity	

Hsp90 inhibitors have received considerable attention for the treatment of advanced cancers [180]. Following drug development in oncology, an increasing number of small molecule inhibitors of Hsp90 have been investigated in neurodegenerative diseases including models of PD (Table 1A & Table 2). Besides many other effects on client proteins and associated pathways, Hsp90 inhibitors induce the activity of the transcription factor HSF-1 and thus lead to increased expression of stress-induced proteins such as Hsp70. The first compound that was investigated in PD models was Geldanamycin, a naturally occurring antibiotic of the Ansamycin family. *McLean et al.* found that treatment with Geldanamycin in cell culture models effectively reduced α-synuclein aggregation through increasing its clearance, leading to reduced toxicity [168]. *Auluck et al.* confirmed neuroprotective effects of Geldanamycin in a *Drosophila melanogaster* model of α-synuclein toxicity [81, 167, 169] and *Shen et al.* found a protective effect in the MPTP mouse model of PD [181]. Interestingly, Hsp90 also seems to be involved in the exocytosis of α-synuclein [171]. Extracellular α-synuclein, once secreted, is subject to endocytosis by adjacent cells and at least a part of the internalized α-synuclein is re-secreted, which could represent a key step in the cascade that allows cell-to-cell propagation of α-synuclein aggregates [49–51]. *Liu et al.* further reported that Hsp90 inhibition with Geldanamycin protects cells against extracellular α-synuclein-induced neurotoxicity by preventing re-secretion of α-synuclein [171]. Although these findings have been encouraging, the use of Geldanamycin has been limited for pharmacokinetic reasons, most importantly its poor solubility and blood–brain-barrier penetration. Other members of the Ansamycin family, like 17-AAG (Tanespimycin) and 17-DMAG (Alvespimycin), have better pharmacokinetic profiles, but other limitations [182]. Similar to Geldanamycin, 17-AAG attenuates α-synuclein toxicity, prevents oligomerization and facilitates α-synuclein clearance in cultured cells [45, 46]. Moreover, 17-AAG can effectively enhance α-synuclein clearance via macroautophagy, a potential key pathway downstream of protein misfolding [173]. Current phase I/II trials for various forms of cancer have demonstrated safety, but the use of 17-AAG in neurodegenerative diseases remains limited because of poor blood–brain-barrier permeability [180]. 17-DMAG displays better solubility but further clinical development of this compound in oncology has not been pursued due to toxicity [180, 183]. In view of these limitations, the clinical utility of all three compounds Geldanamycin, 17-AAG and 17-DMAG is questionable, despite encouraging results in disease models (Table 1A). Novel synthetic small-molecule inhibitors of Hsp90 such as SNX-2112 and derived compounds have been identified through compound library screens for scaffolds that selectively bind the ATP-binding pocket of Hsp90 and display good pharmacokinetic characteristics including blood–brain-barrier penetration. Treatment with SNX compounds in cell culture models of PD resulted in a decrease of both high-molecular weight and monomeric α-synuclein as well as a significant reduction of α-synuclein oligomerization [45] (Table 1A). Despite these promising findings, further *in vivo* evaluation is clearly necessary to evaluate the general prospect of Hsp90 inhibitors for the treatment of synucleinopathies.

**Table 2:** **Pharmacological targeting of molecular chaperones in neurotoxin-induced models of Parkinson’s disease**

Compound	Disease model	Readout	Reference
Geldanamycin	MPTP mouse model	• Chaperone and HSF-1 levels	*Shen et al. 2005*[181]
		• Dopamine levels	
		• Toxicity	
Celastrol	MPTP mouse model	• Hsp70 levels	*Cleren et al. 2005*[184]
		• Dopamine levels	
		• Toxicity	
Trehalose	Epoxomicin cell model	• α-synuclein aggregation	*Casarejos et al. 2011*[185]
		• α-synuclein and chaperone levels	
		• Macroautophagy markers	
		• Proteasome activity	
		• Oxidative stress	
		• Toxicity	
4-phenylbutyrate	Rotenone mouse model	• α-synuclein aggregation	*Inden et al. 2007*[186]
		• α-synuclein levels	
		• Dopamine levels	
		• Behavioral deficits	
		• Oxidative stress	
		• Toxicity	

Modulators of HSF-1 have mainly been evaluated in models of neurodegenerative diseases other than synucleinopathies. For example, Arimoclomol, a compound that prolongs the binding of HSF-1 to heat-shock-response elements and thus increases the expression of Hsp70 and other chaperones under conditions of protein overload, has shown very encouraging results in models of spinal and bulbar muscular atrophy [187] and has even reached clinical testing in amyotrophic lateral sclerosis [188, 189]. Celastrol, a compound that promotes phosphorylation of HSF-1, was found to significantly ameliorate MPTP-induced neurodegeneration in the MPTP mouse model [184] and the DJ-1A *Drosophila melanogaster* model of PD [190] (Table 2). Carbenoxolone (CBX), a glycyrrhizic acid derivative, was found to activate HSF-1 and to promote Hsp70 expression which can ameliorate α-synuclein aggregation in cells [174] (Table 1B).

Given the importance of HSF-1 as the master regulator of chaperone gene transcription and the limitations of global Hsp90 inhibition, small molecules that directly modulate this transcription factor are clearly advantageous. Recently, a yeast-based high-throughput screen for small molecule activators of HSF-1 identified the compound HSF1A. This compound was shown to promote HSF-1 in an Hsp90 independent manner and without the presence of proteotoxicity [191]. HSF1A-mediated Hsp70 induction reduced the *de novo* formation of protein aggregates and ameliorated polyglutamine-induced cytotoxicity in both a cell and *Drosophila melanogaster* model of Huntington’s disease [191]. Another recent sophisticated small molecule screen identified small molecule proteostasis regulators that induce HSF-1-dependent chaperone expression and importantly reduce aggregate formation and toxicity in cells and a *Caenorhabditis elegans* model for expression of expanded polyglutamines [192].

Compounds with direct chaperone activity, or chemical chaperones, are also being evaluated as potential therapies (Table 1C & Table 2). For example, trehalose, a disaccharide, is able to act as a chemical chaperone through direct interaction with client proteins but can also enhance protein clearance via the autophagy pathway, with beneficial effects in different models of major neurodegenerative diseases [175, 176, 185, 193–197]. The chemical chaperones 4-phenylbutyrate [179, 186], mannosylglycerate [178] and most recently mannitol [177] can significantly ameliorate α-synuclein aggregation and toxicity in a variety of PD models including yeast, *Drosophila melanogaster* and mouse models (Table 1C & Table 2). Given the low toxicity of most chemical chaperones tested, these compounds might be good candidates for future drug development.

## Conclusions

Impaired protein metabolism is a unifying theme in neurodegenerative diseases. To prevent the formation of potentially toxic α-synuclein oligomers and aggregates, a number of exciting chaperone-based therapies are under development for use in PD. Encouraging approaches include small molecule inhibitors of Hsp90 and other strategies that target Hsp70 expression or chemical chaperones (Tables 1 & 2). Enhancing chaperone function might be able to prevent early pathological changes such as the formation of α-synuclein oligomers. With the limitations discussed above, a number of studies in disease models clearly implicate a pivotal role for chaperones and protein misfolding in the pathogenesis of PD and other synucleinopathies (Figure 1). It should be cautioned however, that despite promising results in cellular models, *in vivo* data are still limited. The same limitations that apply to all neuroprotective therapies on trial will also challenge testing of chaperone-based therapeutics [17]. It remains a conceptual question, whether a single agent targeted at increasing the expression of chaperone proteins will have an enduring neuroprotective effect given the presence of numerous other established disease pathways and mechanisms [17]. Approaches that employ multiple targets such as the chaperone and proteasome system or chaperones and the CMA pathway seem reasonable. With these and the specific limitations discussed above, it is now on future studies to identify novel approaches capable of preventing α-synuclein misfolding and toxicity in PD and related synucleinopathies.

## Abbreviations

DLB: Dementia with Lewy bodies
ER: Endoplasmic reticulum
Grp78/Bip: Glucose-regulated protein 78
Hsc: Heat shock cognate
HSF-1: Heat shock transcription factor 1
Hsp: Heat shock protein
LAMP-2A: Lysosome-associated membrane protein type 2A
MPTP: 1-methyl-4-pheny-1,2,3,6-tetrahydropyridine
PD: Parkinson’s disease
PERK: Pancreatic-like ER kinase
SIRT1: Sirtuin 1
SNARE: Soluble NSF attachment protein receptor
TAT: Trans-activator of transcription
UPR: Unfolded protein response.
